# Living Alone With Dementia: Challenges, Support, and Paths Forward

**DOI:** 10.1093/geroni/igaf122.880

**Published:** 2025-12-31

**Authors:** Heather Menne, Michael Lepore

## Abstract

Whether due to choice or circumstance, across the globe there are significant numbers of people living alone with dementia in the community. This symposium brings together scholars from the United States and the United Kingdom to explore the experiences of people living alone with dementia, highlighting their unmet needs, well-being, and daily challenges, while emphasizing the need for improved support, policy changes, and community responsiveness. Dr. Kate Singer highlights the increasing number of people living alone with dementia as represented in national surveys, and she presents the rates of people living alone with dementia who report feeling lonely every day or even most days of the week. Dr. Allison Gibson presents on the experiences of individuals living alone with dementia, underscoring their challenges with medication adherence, finances, emergency services, and fear of institutionalization, while also examining their social support, healthcare use, and interest in additional assistance to age in place. Mr. Matt Nelson outlines data from Older Americans Act (OAA) clients who are living alone with dementia, and he shares respondents’ assessments of whether the OAA service helps them remain independent and at home. Dr. Linda Clare presents a scoping review of 150+ unique studies about people living alone with dementia; her results reveal significant unmet needs, social differences, and a lack of targeted support, while recommending policy and practice improvements in service responsiveness, community support, and research inclusivity. To conclude, Dr. Michael Lepore, who has supported policies, programs, and research on people living alone with dementia, offers discussion comments. Alzheimer’s Disease and Related Dementias Interest Group Sponsored Symposium

